# Evaluation of the malaria case surveillance system in KwaZulu-Natal Province, South Africa, 2022: a focus on DHIS2

**DOI:** 10.1186/s12936-024-04873-7

**Published:** 2024-02-14

**Authors:** Maxwell Mabona, Thembekile Zwane, Jaishree Raman, Lazarus Kuonza, Babongile Mhlongo, Poncho Phafane

**Affiliations:** 1 South African Field Epidemiology Training Programme, National Institute for Communicable Diseases, A Division of the National Health Laboratory Service, Johannesburg, Gauteng South Africa; 2 School of Public Health, Faculty of Health Sciences, University of Witwatersrand, Johannesburg, Gauteng South Africa; 3 Centre for Emerging Zoonotic and Parasitic Diseases, National Institute for Communicable Diseases, A Division of the National Health Laboratory Service, Johannesburg, Gauteng South Africa; 4 Wits Research Institute for Malaria Control, Faculty of Health Sciences, University of Witwatersrand, Johannesburg, Gauteng South Africa; 5 UP Institute for Sustainable Malaria Control, University of Pretoria, Pretoria, Gauteng South Africa; 6 KwaZulu-Natal Provincial Department of Health, Pietermaritzburg, KwaZulu-Natal South Africa; 7 Division of Public Health Surveillance, National Institute for Communicable Diseases, A Division of the National Health Laboratory Service, Johannesburg, Gauteng South Africa

**Keywords:** DHIS2, Malaria, Surveillance system, Evaluation, KwaZulu-Natal, Elimination

## Abstract

**Background:**

South Africa set a target to eliminate malaria by 2023, with KwaZulu-Natal (KZN) Province the malaria-endemic province closest to achieving this goal. Objective two of the National Malaria Elimination Strategic Plan (NMESP) focused on strengthening surveillance systems to support the country’s elimination efforts. Regular evaluations of the malaria surveillance systems against the targets of the NMESP objective are crucial in improving their performance and impact. This study aimed to assess whether the malaria surveillance system in KwaZulu-Natal Province meets the NMESP surveillance objective and goals.

**Methods:**

A mixed-methods cross-sectional study design was used to evaluate the malaria surveillance system, focusing on the District Health Information System 2 (DHIS2). The study assessed the data quality, timeliness, simplicity, and acceptability of the system. Key personnel from KZN’s Provincial malaria control programme were interviewed using self-administered questionnaires to evaluate their perception of the system's simplicity and acceptability. Malaria case data from January 2016 to December 2020 were extracted from the DHIS2 and evaluated for data quality and timeliness.

**Results:**

The survey respondents generally found the DHIS2-based surveillance system acceptable (79%, 11/14) and easy to use (71%, 10/14), stating that they could readily find, extract, and share data (64%, 9/14). Overall data quality was good (88.9%), although some variables needed for case classification had low completeness and data availability. However, case notifications were not timely, with only 61% (2 622/4 329) of cases notified within 24 h of diagnosis. During the 5-year study period, the DHIS2 captured 4 333 malaria cases. The majority of cases (81%, 3 489/4 330) were categorized as imported, and predominately in males (67%, 2 914/4 333).

**Conclusion:**

While the malaria surveillance system in KZN Province largely met the NMESP surveillance strategic goals, it failed to achieve the overarching surveillance objective of 100% notification of cases within 24 h of diagnosis. The majority of reported cases in KZN Province were classified as imported, emphasizing the importance of complete data for accurate case classification. Engaging with healthcare professionals responsible for case notification and disseminating aggregated data back to them is needed to encourage and improve notification timeliness.

**Supplementary Information:**

The online version contains supplementary material available at 10.1186/s12936-024-04873-7.

## Background

Malaria continues to rank among the six major causes of death from communicable diseases worldwide [1]. In recent years, there has been a progressive decline in the global burden of malaria, although the pace of reduction has not been as consistent as in previous years [2–4]. Globally, an estimated 231 million cases were reported in 2017, down to 228 million cases in 2018 and 227 million cases in 2019 [2–5]. In 2017, there were an estimated 435 000 deaths from malaria worldwide, which decreased to 405 000 in 2018, but increased to 558 000 in 2019 [2–5]. Malaria's global downward trend from 2017 to 2019 was abruptly reversed in 2020, when there were reported to be 241 million cases and 627 000 fatalities [4]. The World Health Organization (WHO) attributed this sudden increase to the disruption of surveillance activities and malaria control programmes caused by the COVID-19 pandemic, decelerating malaria elimination progress [4].

South Africa is one of the 25 countries identified by the WHO with the potential to eliminate malaria by 2025 [4]. In 2012, South Africa formally adopted an elimination strategy that aimed at eliminating the spread of malaria within its borders by 2018 [6]. Due to financial and logistical challenges, the aim was not achieved [7]. South Africa then set a new malaria elimination goal revising the strategic plan to target elimination by 2023 [8]. However, this new target may also be missed given how the end of 2023 is fast approaching. There were five objectives in the updated National Malaria Elimination Strategic Plan (NMESP), and objective two specifically addresses malaria surveillance [8]. The objective was to strengthen and sustain the surveillance systems to ensure 100% reporting of malaria cases into the Malaria Information System (MIS) within 24 h of diagnosis by 2020 [8]. The strategic goals underneath this objective included having an information system that supplies comprehensive, valid, and timely data [8]. The information system also needs to be easy to use, allowing simple data access, management, and analysis for informed decision-making [8].

In response to this need for an information system that supports elimination efforts, the District Health Information System 2 (DHIS2) was rolled out in South Africa [8–11]. DHIS2 was introduced to help collect routine malaria data, make data flow simple, standardized, cleaner, and allow for efficiency in integrating all data sources required to reach malaria elimination [9–11]. DHIS2 should ideally capture all data necessary to measure progress against set indicators and goals for malaria elimination [9–11]. The WHO considers surveillance as a core intervention in malaria elimination settings and routine information systems as an important surveillance tool [12]. To ascertain whether the programme activities are accomplishing the desired aims and outcomes, the WHO advises that a surveillance system be routinely evaluated [13]. The evaluation of a surveillance system assists in making it more effective, efficient, and impactful [13, 14].

The three malaria-endemic provinces in South Africa (KwaZulu-Natal, Limpopo, and Mpumalanga) all use the DHIS2 as the central system for malaria data and surveillance [15]. Among the three endemic provinces, KZN Province is the closest to elimination, reporting the lowest number of locally-acquired cases between 2013 and 2018 [16]. In an attempt to achieve elimination and sub-national verification, the province's progress is constantly monitored [16]. Inadequate usage, timeliness, and completeness of data from the surveillance system are the main drawbacks that are typically cited by these monitoring activities [16]. Information on whether or not DHIS2's performance in KZN Province meets the set NMESP surveillance objective it was rolled out for is limited. No evaluation has yet been done to rate the DHIS2 surveillance system's use, data quality, timeliness, simplicity, and acceptability in KZN Province.

In light of this background, an evaluation of the malaria case surveillance system in KZN Province focusing on DHIS2 was conducted, to assess its performance and determine if it is meeting the NMESP surveillance objective. The study sought to evaluate the data quality, timeliness, simplicity, and acceptability of the surveillance system. The study findings were expected to identify potential gaps for strengthening and opportunities for improvement. Effective malaria surveillance and information systems are vital in contributing to the province's plans to possibly achieve elimination.

## Methods

### Study setting

**Fig. 1:**
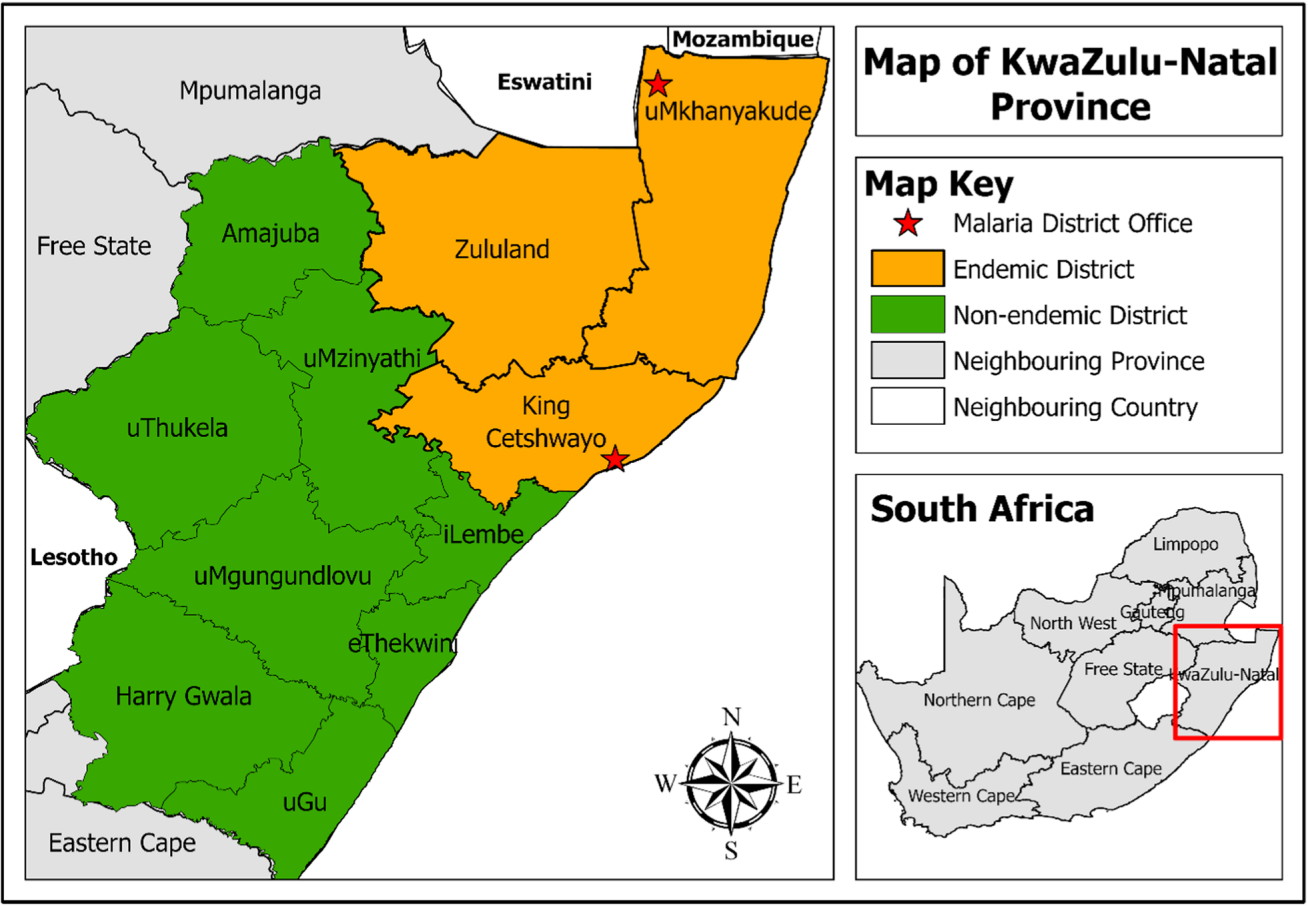
Map of KwaZulu-Natal Province, South Africa, showing the three malaria-endemic districts in the northeast part of the province. Red stars denote the malaria district offices

The study was conducted in the KZN Province of South Africa, focusing on its three malaria-endemic districts; uMkhanyakude, King Cetshwayo, and Zululand (Fig. 1). These three endemic districts are geographically neighbouring each other in the northeast part of the province. This is where the KZN Province shares international borders with Eswatini and Mozambique. Mozambique is estimated to account for approximately 77% of the provinces' malaria cases annually [15, 17]. The three districts have a combined area of about 36 867 KM^2^ and a combined population of approximately 2 552 535 people according to STATS South Africa 2020 population estimates [18]. The province has two official malaria district offices located in uMkhanyakude district (Jozini) and King Cetshwayo district (Richards Bay).

### Malaria surveillance system operations in KZN Province

**Fig. 2:**
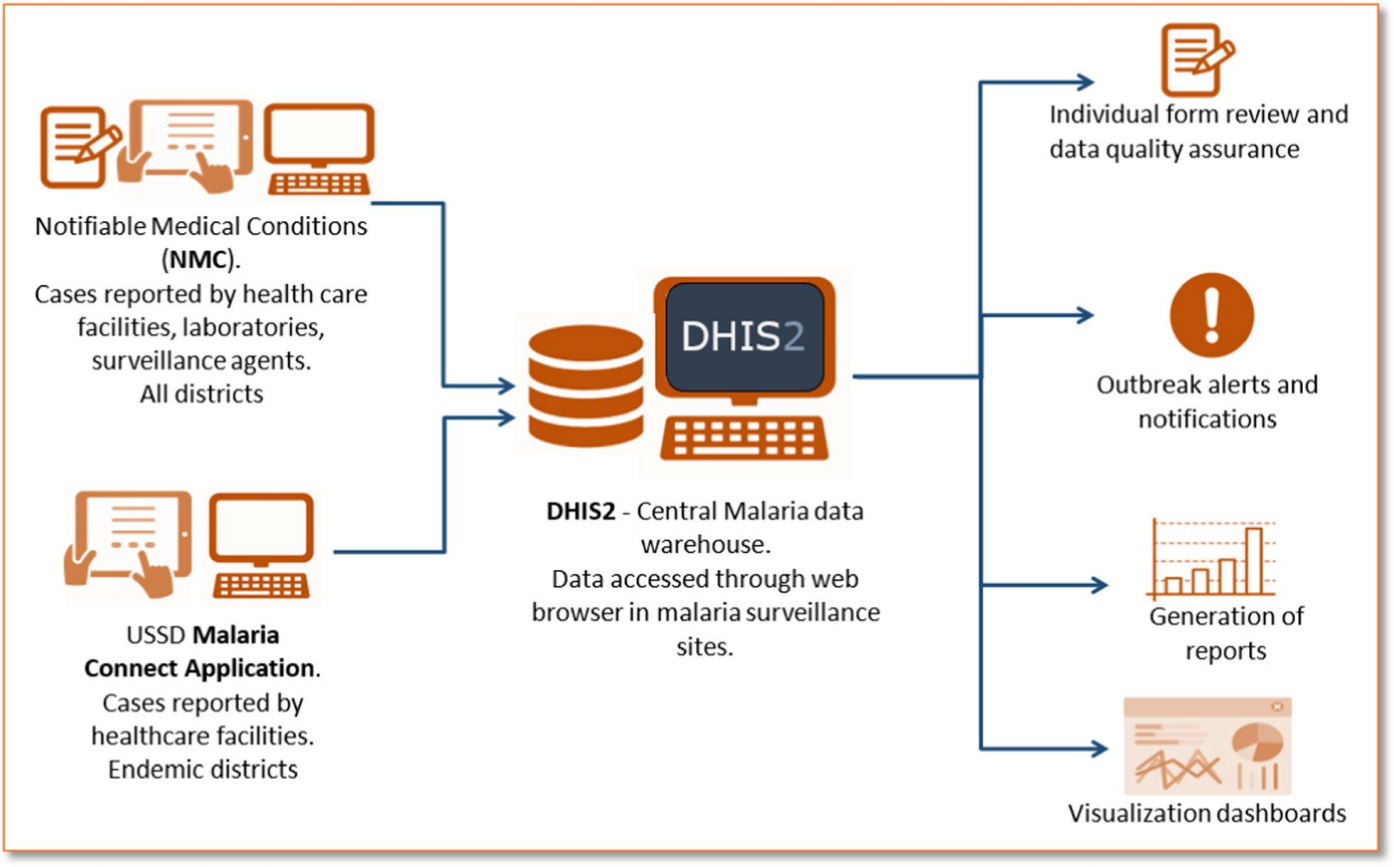
Flow of malaria case data from the Notifiable Medical Conditions application and Malaria Connect into the District Health Information System 2 (DHIS2), in KwaZulu-Natal Province, South Africa

Malaria data collection, specifically the testing and diagnosing of cases at a patient level and reporting is typically done by healthcare workers and malaria surveillance officers. Healthcare workers in malaria-endemic districts notify malaria cases using a USSD-based Malaria Connect application via mobile phones free of charge. They are also obliged by the National Health Act (61 of 2003) to report the cases on the Notifiable Medical Conditions (NMC) application [19]. Malaria surveillance officers also actively go to communities, farms, taxi ranks, and border posts within endemic districts to screen and test people for malaria. Lastly, reporting is also done by laboratories using the NMC application to report specimens that tested positive for malaria parasites. The NMC application is used for reporting by both malaria-endemic and non-endemic districts. Ideally, all cases notified at a patient level through the NMC application or Malaria Connect should be transferred directly or indirectly into DHIS2 (Fig. 2).

### Study design

A mixed-methods cross-sectional study design was employed utilizing primary and secondary data. Primary data were collected through an online survey among key personnel of the surveillance system and used to evaluate simplicity and acceptability. Secondary malaria case data extracted from the DHIS2 were used to evaluate data quality and timeliness. These four attributes were assessed using the updated Centre for Disease Control and Prevention (CDC) Guidelines for Evaluating Public Health Surveillance Systems [20].

### Study population

The study population for the online survey were all available key personnel involved in surveillance. This included the programme managers, case investigation officers, malaria information officers, environmental health practitioners, entomologists, and data capturers. The study characterized these cadres as key personnel because they routinely work with DHIS2, have access to data, and work at provincial and district levels of surveillance where all malaria data from facilities are reported.

For the secondary data analysis of confirmed malaria cases, the study population were all the malaria cases that met the case definition. A case was defined as a patient recorded on the DHIS2, who had malaria confirmed through a rapid diagnostic or laboratory test between January 2016 and December 2020. Malaria cases on DHIS2 are classified as either local, locally imported, or imported. Imported cases are defined as cases that were contracted outside the border of South Africa. Local cases are cases reported in endemic areas where local transmission cannot be ruled out. Locally imported cases are cases detected in non-endemic areas where there is a history of travel to endemic regions in South Africa or in endemic areas where there is proof that the infection was contracted in a different endemic area.

### Sampling method

Survey respondents were selected using convenience sampling. This was by including key personnel who use and know the surveillance system, were available during the data collection period, and gave consent to respond. Respondents were provided with a study information sheet (Additional file 1), and a consent form (Additional file 2) to sign before receiving a questionnaire. For the secondary data analysis of the malaria-diagnosed cases, all cases from January 2016 to December 2020 were included in the analysis.

### Data collection

The Research Electronic Data Capture (REDCap) web application was used to develop and distribute a self-administered questionnaire (Additional file 3) [21]. The questionnaire contained Likert-scale and multiple-choice questions which collected respondents’ socio-demographic information and the respondent’s perception of the surveillance system. The Likert-scale questions were developed on a five-point Likert-scale ranging from strongly disagree to strongly agree. Respondents received the REDCap questionnaires by email, and they had four weeks to reply. Three automatic email reminders were sent out via REDCap after each week of no response to those who had not yet responded. After the third reminder, non-responders were reminded telephonically. After the telephonic reminder and the conclusion of the four-week data collection period, failure to respond was taken to indicate reluctance to participate. The secondary malaria case data for the KZN Province from 2016 to 2020 were requested from the routinely collected data on the DHIS2.

### Data management

The responses from the questionnaires captured using REDCap were checked for errors and inconsistencies at the end of data collection. The secondary malaria case data were received in a Microsoft Excel format. The data were received without patient identifiers; new unique anonymous identifiers were created for analysis purposes. Data cleaning and management of both data sets included looking for and dropping duplicates, renaming, and labelling variables. The data sets were then imported to STATA statistical software (Version 17) where data analysis was done.

### Data analyses

Summary statistics were used to describe the characteristics of survey respondents and malaria-diagnosed cases. Medians with interquartile ranges were used for continuous variables and percentages, frequency tables, and graphics for categorical variables. The malaria control programme team picked the four attributes of the surveillance system (data quality, timeliness, simplicity, and acceptability) as the primary attributes to be evaluated. Data quality was assessed by looking at three key components of the secondary DHIS2 data; completeness, availability, and validity. A completeness percentage was calculated by finding the proportion of fields in selected variables that were completed, i.e. not left blank. Fields containing the words “unknown” or “unavailable” were considered complete, hence a ‘data availability percentage’ was also calculated. The data availability percentage was calculated by finding the proportion of fields per variable for which the field was not left blank, written “unknown”, “unavailable” or “null”. A validity percentage was calculated by finding the proportion of fields per variable without information filled out incorrectly, incompletely, or with error. Additionally, among the recorded fields, the field couldn't contain information that was out of range or invalid (such as the date of 31 February, an age of 523 years, or a symptom under a gender variable). The completeness, availability, and validity percentages were all calculated using the total number of fields or observations in the data set as the denominator. Overall data quality was considered good if these percentages had a combined average above 80%.

Assessment of timeliness involved evaluating the amount of time taken between the diagnosis of a malaria case and notification on the surveillance system. The difference in days between the date of diagnosis and the date of notification was calculated for each case. Since malaria is a category one notifiable medical condition in South Africa that requires notification within 24 h, cases notified after 24 h were considered untimely notifications. Overall timeliness was considered good if 100% of the cases were notified timely as per the NMESP targets.

Simplicity was assessed objectively through the online survey to determine the complexity of data collection methods, capturing, analyses, and dissemination of reports. The simplicity of case definitions and the ease of obtaining surveillance data for generating reports were also assessed. Acceptability was also assessed objectively by checking the level of willingness of respondents to continue consistently inputting data, and extracting reports from the surveillance system. For both simplicity and acceptability, the responses for each of the five-point Likert-scale questions were analysed and presented using bar graphs. The multiple-choice questions were analysed and presented using counts, and percentages. Where a question allowed respondents to ‘specify other’, all the responses were quoted as is. Overall, if the vast majority of respondents strongly agreed or agreed, then the results of the questions gauging performance were deemed to be simple and/or acceptable.

## Results

### Descriptive analysis of the malaria-diagnosed cases

A total of 4 333 malaria cases were retrieved from the DHIS2 from January 2016 to December 2020 (Additional file 4). Males accounted for more than two-thirds of the cases (67.3%, 2 914/4 333). The median age of the cases was 26 years (inter-quartile range (IQR): 19–36 years). The majority of the cases had unknown citizenship (59.7%, 2 585/4 333), followed by cases who had a citizenship of Mozambique (29.0%, 1 258/4 333), and then South Africa (9.3%, 401/4 333). The majority (80.6%, 3 489/4 330) of the notified cases were classified as imported cases, while over 8% (365/4 330) of cases were unclassified (Table 1).

**Table 1:** Socio-demographic characteristics of malaria-diagnosed cases in KwaZulu-Natal Province from the DHIS2, January 2016 to December 2020

Socio-demographic characteristics of malaria-diagnosed cases		Frequency (n) Total: 4 333	Percentage (%)
Sex	Male	2 914	67.3
	Female	1 419	32.7
Age*	0–9	585	13.5
	10–19	559	12.9
	20–29	1 416	32.7
	30–39	945	21.8
	40–49	474	10.9
	50 and above	353	8.2
Citizenship	Mozambique	1 258	29.0
	South Africa	401	9.3
	Malawi	21	0.5
	Ethiopia	14	0.3
	Zimbabwe	14	0.3
	Unknown	2 585	59.7
	Other countries	40	0.9
Case classification*	Imported	3 489	80.6
	Local	429	9.9
	Locally imported	49	1.1
	Unclassified	363	8.4

^*^Observations without a record were excluded. *n* number of malaria cases

**Fig. 3:**
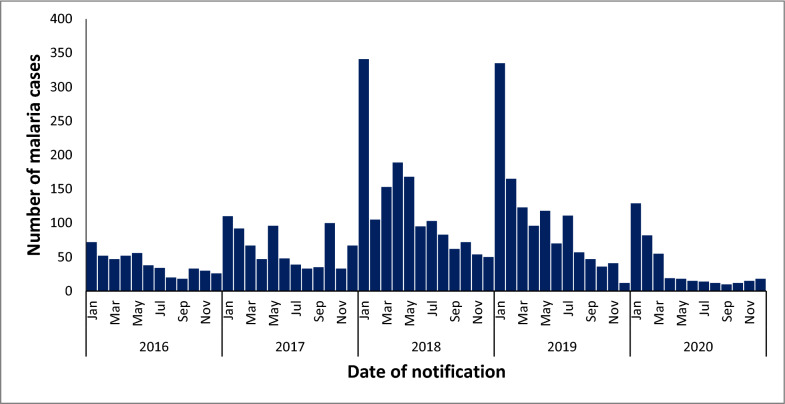
An epidemic curve of malaria-diagnosed cases from the DHIS2 by date of notification in KwaZulu-Natal Province, January 2016 to December 2020

Over the 5-year study period, the number of notified malaria cases increased from 478 in 2016 to 767 in 2017 (38% increase). The yearly increase continued into 2018 when 1 475 cases were notified (48% increase). The years 2018 and 2019 were the peak of malaria notifications over the five years with January being the month where the highest number of cases were seen. Cases peaked between September and May of 2017/2018, and 2018/2019 with 1 191 and 1 075 cases, respectively. There was however a decline in cases after these two peaks with the period between September and May of 2019/2020 having only 439 cases notified (59% decrease) (Fig. 3).

### Descriptive analysis of survey respondents

Among the 18 key personnel invited, 78% (14/18) responded. More than half (57%, 8/14) of the respondents were female, and half (50%, 7/14) were environmental health practitioners. While some respondents reported one primary work area, some reported rotating between two work areas. A total of 11 respondents reported uMkhanyakude district as one of their primary work areas. More than half (57%, 8/14) of respondents worked in a malaria district office, with all of them reporting the Jozini office (uMkhanyakude district). Thirty-one percent (4/13) of the respondents had been using the DHIS2 for less than a year, 62% (8/13) for 1–2 years, and only 8% (1/13) for 3–4 years. Most (71%, 10/14) respondents reported that they had been trained on how to use the DHIS2 (Table 2).

**Table 2:** Socio-demographic characteristics of respondents interviewed during a malaria surveillance system evaluation in KwaZulu-Natal Province, 2022

Socio-demographic characteristics of respondents		Frequency (n) Total = 14	Percentage (%)
Sex	Male	6	43
	Female	8	57
Occupation	Programme manager	4	29
	Case investigation officer	0	0
	Malaria information officer	0	0
	Environmental Health Practitioner	7	50
	Data capturer	2	14
	Entomologist	1	7
Primary workplace	UMkhanyakude district	11	61
	King Cetshwayo district	1	6
	Zululand district	4	22
	Provincial offices	2	11
Working in a malaria district office	Jozini	8	100
	Richards Bay	0	0
Number of years using DHIS2	Less than 1 year	4	31
	1–2 years	8	61
	3–4 years	1	8
	5 years and above	0	0
Trained on DHIS2 use	Yes	10	71
	No	4	29

DHIS2 = District Health Information System 2. *n* number of respondents

### Evaluation of performance attributes

#### Data quality

Data quality refers to the level of completeness, validity, accuracy, conformity, and consistency of the data. Overall, most variables assessed had the majority of fields completed. The percentage of completed fields however varied across the different variables. Variables like sex, age, and notification date had almost all (99–100%) fields completed, while variables like citizenship (40.3% completion), symptoms (42.5% completion), and residential address (67.6% completion) had lower percentages of completed fields. Even though some fields were not left blank, they had no data available as they were filled with “unknown”. When calculating the percentage of fields per variable that had actual data, the results also varied. Variables like sex, age, and notification date had less than 0.1% of fields filled with “unknown”. On the other hand, only 41.6% of fields under symptoms and 63.5% of fields under travel history had information, the rest of the fields were blank or labelled “unknown”.

Despite completeness and data availability being lower for some variables, the validity of all the variables was almost without error. Among all the fields that were completed, no fields were filled out with values outside of allowed ranges, with errors, or with invalid values. All responses for categorical variables were of the predetermined categories for that variable, for instance, all responses given for sex were male, female or unknown, and no other entries unrelated to sex were found. Similarly, all responses for numerical variables and dates fell within acceptable ranges and were filled out using numbers (Table 3). The completeness, availability, and validity percentages of all the selected variables had a combined average of 88.9%.

**Table 3:** Completeness, data availability, and validity of selected variables for the evaluation of the malaria surveillance system in KwaZulu-Natal Province, January 2016 to December 2020

Selected variables	Completeness (%)	Data availability (%)	Validity (%)
Sex	100	100	100
Age	99.9	99.9	100
Symptoms	42.5	41.6	100
Travel history	98.4	63.5	100
Case classification	98.9	91.6	100
Symptom onset date	96.8	96.8	100
Notification date	99.9	99.9	100
Residential address	67.6	67.6	100
Treatment given	80.5	79.9	100
Citizenship	40.3	100	100
Mean percentage (%)	82.5	84.1	100

#### Timeliness

**Fig. 4:**
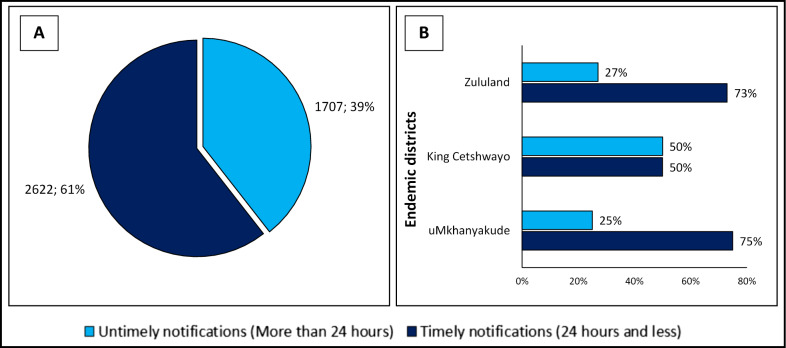
Percentages of malaria cases notified timely and untimely, KwaZulu-Natal Province, January 2016 to December 2020

Timeliness refers to the time taken between steps in the surveillance system. Of the 4 332 cases that had both diagnosis and notification dates available, three cases (0.1%) were excluded as they had a notification date that was before the diagnosis date. Of the remaining 4 329 cases, 60.6% (2 622/4 329) were notified/reported timely (within 24 h of diagnosis) while the other 1 707 (39.4%) cases were reported/notified untimely (Fig. 4A). The majority of the untimely reported/notified cases (83%) were notified within seven days of diagnosis. Amongst all the 4 329 cases, the median number of days taken to notify a case was one day (IQR: 0–3 days). The timely notification rate in the three endemic districts ranged from 50 to 75% (Fig. 4B).

#### Simplicity

**Fig. 5:**
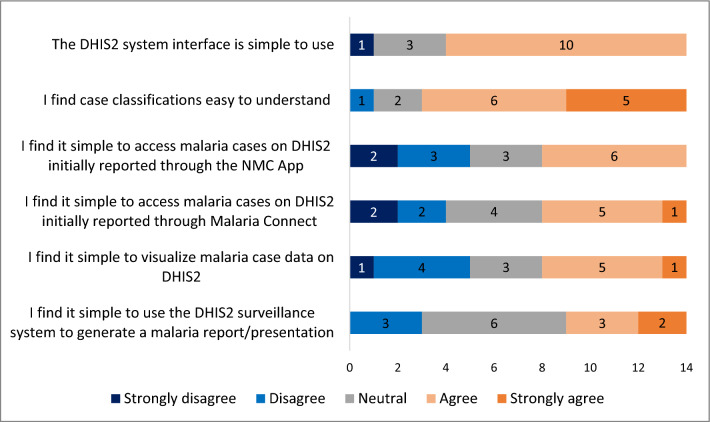
Perceived simplicity of DHIS2 by key personnel through 5-point Likert-scale questions, KwaZulu-Natal Province, 2022

Simplicity refers to how simple the structure of the surveillance system is, and the ease of use of the system. The majority (71%, 10/14) of the respondents agreed that the DHIS2 interface was simple to use (Fig. 5). However, when asked if there were aspects of the interface that can be improved, 64% (9/14) of the respondents also agreed, stating that the interface's loading speed and the simplicity of navigating could be enhanced. Over half (64%, 9/14) of the respondents agreed that they find it simple to share, transfer, enter, edit, and store data on DHIS2. The remaining respondents (36%, 5/14) who did not find it simple stated having challenges with entering, accessing, editing, sharing, transferring, and saving data. Two respondents went on to elaborate and say:

*“I need more training on how to use DHIS2”*

*“I can’t share data out of DHIS2, to Word, PowerPoint or PDF”*

Furthermore, 79% (11/14) of the respondents agreed that they found case classifications (local case, imported case, locally imported case, indigenous case) easy to understand. Three respondents expressed having difficulties understanding how to classify cases as ‘locally imported’ or ‘imported’.

Only six (43%; 6/14) respondents agreed that it was easy to find cases on DHIS2 initially reported through the NMC application or the Malaria Connect notification system. When respondents were asked if they found it simple to visualize malaria data on DHIS2 through pivot tables, graphs, and maps, 36% (5/14) felt that they did not. After probing for the challenges experienced when visualizing data, one respondent stated:

*“It is time-consuming, complicated, and it is not easy to understand the visualized data”*

Lastly, only 36% (5/14) of the respondents agreed that they find it simple to use the DHIS2 surveillance system to generate a report or presentation.

#### Acceptability

**Fig. 6:**
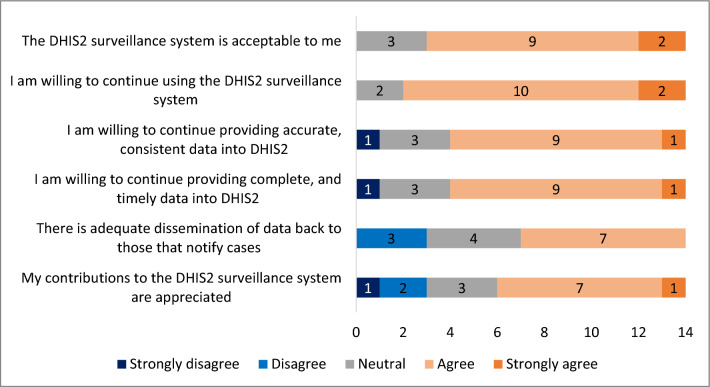
Acceptability of DHIS2 by key personnel through 5-point Likert-scale questions, KwaZulu-Natal Province, 2022

Acceptability refers to the willingness of users and stakeholders to participate in the surveillance system. The majority (79%, 11/14) of the respondents agreed that the DHIS2 surveillance system is acceptable to them (Fig. 6). Above 80% (86%, 12/14) indicated willingness to continue using the system. Seventy-one percent (10/14) of the respondents also agreed that they were willing to continue providing accurate, consistent, complete, and timely data. However, only seven (50%, 7/14) respondents agreed that there was adequate dissemination of aggregated data back to those who notify cases, with only eight (57%, 8/14) respondents of the opinion that their contributions to the surveillance system were appreciated by the surveillance team and/or control programme. Of the five respondents who had suggested system improvements, three (60%) stated that their improvement suggestions were incorporated into the system.

## Discussion

The study evaluated the performance of the malaria case surveillance system in KZN Province with a focus on DHIS2 to determine if the system met the NMESP surveillance objective. The results demonstrated that the DHIS2 data were of good quality overall, notwithstanding some variables' incompleteness and low data availability. The study found timeliness to be below set targets, as only 61% of the cases were notified within 24 h of diagnosis. With some aspects to improve, key personnel of the system generally found DHIS2 acceptable, easy to use, and that they could readily find, extract, and share data. The descriptive analysis part of the study showed that the KZN Province saw a decline in notified cases in 2020 and that imported cases were predominant over the study period.

Overall, the data on DHIS2 for the five years were of good quality, with a combined average of 88.9%. Although some variables, such as citizenship, residential address, symptoms, and travel history, had low completeness and data availability percentages, other variables such as sex and age offered high-quality data. The data's validity was high since almost all records fell within expected ranges, were free of grammatical or spelling errors, and represented each variable's expected record. This might be a result of DHIS2's automated data entry process, in which most fields are filled in by selecting from a drop-down menu rather than manual typing input. These findings were similar to an evaluation of DHIS2 malaria data conducted recently in Ghana [22]. In their evaluation, the quality of the data was rated 95% also using the CDC guidelines for surveillance system evaluation [22]. It is important to note that poor data availability and completeness for certain variables can have a knock-on negative effect on other variables. Without detailed travel histories, accurate case classifications are not possible, and this may have contributed to the relatively high number of unclassified cases detected in this study. The need for complete and accurate data is emphasized by one strategic goal under the surveillance objective of the NMESP [8]. This is because complete and accurate data for each case supports correct, prompt case investigation and classification.

The study found that even though 61% of cases were notified timeously, this was below the NMESP timeliness target of notifying 100% of the cases within 24 h of diagnosis [8]. This finding is similar to an evaluation done in Nigeria were for earlier years, timeliness was below 70% [23]. However, other malaria studies conducted in Ghana, Yemen, and Nigeria had over 90% of cases reported timely, although the malaria burden in these settings may be different from this study’s [22, 24, 25]. Timely notification of malaria cases allows prompt, informed response and outbreak prevention [8].

The study found that the malaria case surveillance system in KZN Province was simple. The data flow pathways were clear and easy to follow. The majority of the key personnel found the DHIS2 interface simple to access and use. This is similar to the findings of a malaria surveillance evaluation conducted in Nigeria where the system was found to be simple [23]. However, there were suggestions that the ease of navigation, the loading speed, and the case classification process could be improved. Distinguishing locally imported cases from imported cases was flagged as being challenging by a small number of respondents. This differs from the findings of similar evaluations conducted in Nigeria where case definitions and classifications were found to be simple to understand and use by participants [23, 25]. Incorrectly classified cases might undermine efforts to meet the NMESP indicator of measuring the proportion of local to imported cases correctly classified. It should be noted that not all personnel interviewed are required to classify cases.

Other factors that affected the overall simplicity of the system were data visualization and finding cases. Simple user-friendly visualization of malaria data is crucial for pragmatic decision-making. The NMESP states that DHIS2 data should be visualized, analysed, and used for improved operational programme planning [8]. The DHIS2 dashboard was designed to allow users to easily visualize data and generate reports automatically when needed [9]. The study found that visualizing data on DHIS2 and using it for reports and decision-making was not simple enough. In addition, cases initially reported through the NMC application and Malaria Connect were not easily found on DHIS2. This is despite the systems’ data flow design that is supposed to facilitate the direct or indirect import of cases reported through the two data sources into the DHIS2 system.

The findings of this study showed that the surveillance system was overall acceptable. The majority of the key personnel were willing to continue providing consistent, timely, and accurate data. This was similar to the findings of other malaria surveillance evaluations in Africa and the Middle East [23–25]. Conflicting with these studies, the evaluation showed that not many respondents felt like their contributions to the surveillance system were appreciated. Another factor that decreased the acceptability of the surveillance system was information feedback to lower surveillance levels. There seemed to be inadequate dissemination of aggregated data back to those who notify cases. These were also the findings of a study that assessed the performance of malaria surveillance systems in 16 countries including South Africa [26]. Feedback to lower surveillance levels is crucial for the continuation of reporting, staff motivation, and overall acceptability [27].

This study showed that the KZN Province saw a remarkable decline in notified cases in 2020 compared to the four preceding years. The decline in notified cases in 2020 was also noted country-wide. The National Institute for Communicable Diseases (NICD) reported that this might have been attributable to COVID-19-related movement restrictions and malaria being misdiagnosed as COVID-19 [1, 28, 29]. This drop in cases is however contrary to the findings of the WHO 2021 malaria report. The report showed an increase in malaria cases globally in 2020 [4]. The WHO attributed the global increase to disruptions in malaria surveillance, control, and prevention programmes caused by the COVID-19 pandemic [4]. These disruptions in surveillance may have led to an underreporting of cases in the KZN Province, hence the observed decline may not be a true reflection of prevalence.

The study also revealed that the majority of cases were imported from outside the borders of South Africa. This has been the trend for several years according to previously conducted studies in the KZN Province [15, 16, 30]. One study that looked at malaria cases from 2008 to 2018 showed that less than a third of them were indigenous, with the larger proportion being cases that were infected in neighbouring countries, mostly while traveling [15]. This emphasizes the need for the surveillance system to continue offering high-quality data especially for variables like travel history to allow these cases to be classified as imported, and not local. An accurate count of local cases particularly, is important for the KZN Province as the goal of elimination can only be achieved by reducing the number of local cases to zero.

This study had some limitations. The limited time available for data collection could have affected the response rate and compromised the representativeness of the surveillance personnel. Only key personnel at the provincial and district levels of malaria surveillance who utilize DHIS2 were included in this study. Healthcare workers at the healthcare facility and laboratory levels, who are responsible for identifying, diagnosing, and notifying cases but do not have direct access to DHIS2, were not included. However, their perspectives are essential in understanding why some cases are not promptly notified and they could have provided insights into the actual levels of aggregated data dissemination back to them.

In addition, the study focused on evaluating the surveillance system concentrating on DHIS2. It would be valuable to conduct an evaluation that examines malaria-diagnosed cases for the same period from Malaria Connect and the NMC system. This would help determine whether all cases from these two data sources are integrated into DHIS2, and if regular data reviews are conducted to correct variances. Furthermore, such an evaluation could also assess the flexibility and adaptability of the surveillance system to changes such as the COVID-19 pandemic, and the implementation of the NMC mobile application.

## Conclusion

The majority of the strategic goals under the surveillance objective of the NMESP are being successfully met by the KZN Province malaria surveillance system. This includes providing data that is of good quality, valid, simple to access, visualize and use for decision-making. The components of the surveillance system were user-friendly with simple data flow and an easy-to-use DHIS2 interface. Users should be encouraged to complete the self-training courses and watch training videos on DHIS2. This can improve efficiency in tasks like data entering, visualization, extraction, transferring, storing, and generating reports. Simple case classification and sub-classification algorithms should also be made readily available to all personnel, especially those who have roles that may require them to classify cases. Despite good performance on most surveillance strategic goals, the surveillance system did not meet the overarching surveillance objective (objective two) of the NMESP. To guarantee 100% reporting of malaria cases within 24 h, there is still much work to be done. Engagements with all who notify cases at a patient level are needed to find reasons for delayed case notification. Feedback of aggregated data to them also needs to improve to encourage timely notification. Addressing these gaps in the surveillance system is vital in contributing to the province's plan to possibly achieve malaria elimination.

### Supplementary Information

**Additional file 1: **Study information sheet.

**Additional file 2: **Participant/respondent consent form.

**Additional file 3: **Key personnel questionnaire.

**Additional file 4: **Secondary data management flow chart.

## Data Availability

The secondary data are publicly available through the National District Health Information Software 2 online database at https://gp.dhis.dhmis.org/. The primary data are also available from the corresponding author upon reasonable request.

## Abbreviations

NMESP: National malaria elimination strategic plan
MIS: Malaria information system
NMC: Notifiable medical conditions
DHIS2: District health information system 2
CDC: Center for disease control and prevention
USSD: Unstructured supplementary service data
REDCap: Research electronic data capture
DoH: Department of health
WHO: World Health Organization
KZN: KwaZulu-Natal
SA: South Africa
