# Supplementary material for: Evaluation of the malaria case surveillance system in KwaZulu-Natal Province, South Africa, 2022: a focus on DHIS2
Source: Malar J. 2024 Feb 14;23:47. doi: 10.1186/s12936-024-04873-7 (PMC10865712; doi:10.1186/s12936-024-04873-7)
Supplement: Supplementary file 1 — Additional file 1: Study information sheet. [file 12936_2024_4873_MOESM1_ESM.docx]

**Supplementary information document**

**Additional file 1: Study information sheet**

**Study title: Evaluation of the Malaria Case Surveillance System in KwaZulu-Natal Province, South Africa, 2022: A focus on DHIS2**

**Greetings:** Good day, my name is Maxwell Mabona, a South African Field Epidemiology Training Programme resident and Master’s student from the University of the Witwatersrand medical school (School of Public Health).

**Introduction**: We are conducting a research study to evaluate the malaria case surveillance system (DHIS2) used in KwaZulu-Natal. This is in accordance with the World Health Organization, which recommends that a surveillance system should be reviewed periodically to determine whether the program activities are functioning effectively and they are achieving the desired outcomes. The study aims to see if the malaria case surveillance system (DHIS2) is meeting the goals underneath objective two of the National Malaria Elimination Strategic Plan (NMESP).

**Invitation to Participate**: We would like to invite you to participate in this research study. Your participation will involve answering an online short self-administered questionnaire designed to gather information for the study.

**What is involved in the study:** If you agree to take part in this study, you will be participating in a self-administered online questionnaire which will take about 15 minutes of your time. The questionnaire is targeting KwaZulu-Natal’s malaria program managers, case investigation officers, malaria information officers, environmental health practitioners, and data captures of the malaria case surveillance and reporting system.

**Risks of being involved in the study:** There are no foreseen risks involved in participating in this study.

**Benefits of being in the study:** There are also no direct individual benefits of being involved in the study. However, the overall benefits of the study are that the results will be used to make recommendations to strengthen and improve the surveillance system if needed. You will be given pertinent information on the study while involved in the project and after the results are available.

**Participation is voluntary:** Your participation in the survey is voluntary and there will be no negative consequences if you do not wish to participate. You are free to withdraw/drop out of the study at any time. You will not be required to provide a reason for withdrawing and any data you already provided will in default be destroyed, unless the you specifically consent to its retention. If you have any questions, do not hesitate to ask.

**Reimbursements:** Participation in the research will not cost you any money nor will they be any payments for participation.

**Confidentiality:** Responses will completely be confidential and anonymous. The results will not be linked to individual respondents. The data will be password-protected and only accessible to the principal researcher. All data collected in the course of the study will be securely retained for two (2) years, if a scientific publication arises from the study and six (6) years, if there is no publication. Thereafter it will be destroyed accordingly.

**Anonymity:** You will not be required to provide any personal or identifying information such as your name or identity number, responses will completely be anonymous.

**Contact details of researchers:**

Maxwell Mabona, [MaxwellM@nicd.ac.za](mailto:MaxwellM@nicd.ac.za)

033 940 2432 / 079 919 8332

**Supervisor:**

Mrs. Moshibudi Poncho Phafane, ponchob@nicd.ac.za / [Poncho.Bapela@kznhealth.gov.za](mailto:Poncho.Bapela@kznhealth.gov.za)

033 940 2434

**Outputs:** The outputs of the research study will be an evaluation report. A copy of the participant information document, a summary of your responses, and the results of the study (evaluation report) once available is available upon request.

**Contact details of HREC administrator and chair**: This study has been approved by the Human Research Ethics Committee (Medical) of the University of the Witwatersrand, Johannesburg (“Committee”). A principal function of this Committee is to safeguard the rights and dignity of all human subjects who agree to participate in a research project and the integrity of the research.

If you have any concern over the way the study is being conducted, please contact the Chairperson of this Committee who is Professor Clement Penny, who may be contacted on telephone number 011 717 2301, or by e-mail on Clement.Penny@wits.ac.za. The telephone numbers for the Committee secretariat are 011 717 2700/1234 and the e-mail addresses are Zanele.Ndlovu@wits.ac.za and Rhulani.Mukansi@wits.ac.za

Thank you for reading this Study Information document.

**Date:** August 2022
