# Supplementary material for: Evaluation of the malaria case surveillance system in KwaZulu-Natal Province, South Africa, 2022: a focus on DHIS2
Source: Malar J. 2024 Feb 14;23:47. doi: 10.1186/s12936-024-04873-7 (PMC10865712; doi:10.1186/s12936-024-04873-7)
Supplement: Supplementary file 2 — Additional file 2: Participant/respondent consent form. [file 12936_2024_4873_MOESM2_ESM.docx]

**Additional file 2: Participant/respondent Consent Form**

**Study title: Evaluation of the Malaria Case Surveillance System in KwaZulu-Natal Province, South Africa, 2022: A focus on DHIS2**

**Consent**

I have been provided with a study information sheet that explains the nature and processes involved in this study; I was given time to read it, in the language I best understand. I was given time to ask any questions I wanted to and found any answers given to me to be reasonable and satisfactory. I believe I fully understand why the study is being conducted and what the intended outcomes will be. I understand that there will be no immediate benefit to me, should I agree to participate, nor will I receive any payment; conversely, participation will not cost me anything but my time. I understand that, even if I initially consent to take part in the study, I may subsequently withdraw at any time and would not be required to give any reasons. I have been given a range of contact details, listed below. If I require further information or become concerned about any aspect of this study, I am free to speak to any of these contacts.

I, therefore, give my consent to participate in this research study: No **☐** Yes **☐**

**Signature or mark:** _______________________________

**Date:** __________________________________________

**Place:** _________________________________________
