# Supplementary material for: Evaluation of the malaria case surveillance system in KwaZulu-Natal Province, South Africa, 2022: a focus on DHIS2
Source: Malar J. 2024 Feb 14;23:47. doi: 10.1186/s12936-024-04873-7 (PMC10865712; doi:10.1186/s12936-024-04873-7)
Supplement: Supplementary file 3 — Additional file 3: Key personnel questionnaire. [file 12936_2024_4873_MOESM3_ESM.docx]

**Additional file 3: Key Personnel Questionnaire**

**Evaluation of the Malaria Case Surveillance System in KwaZulu-Natal Province, South Africa, 2022: A focus on DHIS2**

Date Survey was completed (DD/MM/YYYY): ____ ______ ________

**Section 1- Socio-demographic details**

1. Please indicate your gender:

Male **☐** Female **☐**

1. What is your current position or occupation?

- Malaria Programme Manager **☐**
- Case investigation officer **☐**
- Malaria information officer **☐**
- Environmental Health Practitioner **☐**
- Data Capturer **☐**
- Other **☐** Please specify____________________________

1. Where are you normally working from?

- UMkhanyakude District **☐**
- King Cetshwayo District **☐**
- Zululand District **☐**
- Provincial offices **☐**
- Other District (Non-endemic) **☐**

1. Do you work in a malaria surveillance office/sentinel site?

Yes **☐** No **☐**

If yes, please indicate the surveillance sentinel site:

Jozini **☐**  Richards Bay **☐**

1. How long have you been using DHIS2 (in years)?

Less than 1 year **☐** 1-2 years **☐** 3-4 years **☐** 4-5 years **☐** >5years **☐**

1. Have you ever been trained on how to use DHIS2?

Yes **☐** No **☐**

If yes, please indicate the number of times have you been trained on how to use DHIS2? ________

And if yes, please indicate the year you were last trained on DHIS2: (YYYY) _________

**Section 2- System Attributes**

**Simplicity**

1. Is it simple to access and log into the DHIS2 system? Rate from 1 – 5

(1 = Strongly disagree, 2 = disagree, 3 = Neutral, 4 = Agree, 5 = Strongly agree)

___________________

1. Do you consider the DHIS2 surveillance system interface simple to use? Rate from 1 – 5

(1 = Strongly disagree, 2 = disagree, 3 = Neutral, 4 = Agree, 5 = Strongly agree)

___________________

1. Do you find it simple to share, transfer, enter, edit, and store data on DHIS2?

Yes **☐** No **☐** If no, please indicate the area that usually gives you a challenge (select all that apply):

- Entering data **☐**
- Accessing data **☐**
- Editing data **☐**
- Sharing data **☐**
- Transferring data **☐**
- Saving/ storing data **☐**
- Other **☐** Please briefly specify______________

1. Are there aspects of the DHIS2 surveillance system interface that need improvement for it to be more user-friendly?

Yes **☐** No **☐** If yes, please indicate the aspect that should be improved (select all that apply):

- Logging in and out **☐**
- Ease of navigation **☐**
- Loading speed **☐**
- Other **☐** Please briefly specify______________

1. Do you find the case definitions/classifications (local case, imported case, locally imported case, indigenous case) easy to understand? Rate from 1 – 5

(1 = Strongly disagree, 2 = disagree, 3 = Neutral, 4 = Agree, 5 = Strongly agree)

___________________

1. Do you ever find it challenging to classify a malaria case according to the case definition/classification?

Yes **☐** No **☐** If yes, please indicate the type of cases that usually give you a challenge classifying (select all that apply):

- Local cases **☐**
- Imported cases **☐**
- Locally imported cases **☐**
- Indigenous cases **☐**
- Other **☐** Please briefly specify______________

1. Do you find it simple to access malaria cases on DHIS2 that were initially reported through the Notifiable Medical Conditions application (NMC App)? Rate from 1 – 5 (1 = Strongly disagree, 2 = disagree, 3 = Neutral, 4 = Agree, 5 = Strongly agree)

___________________

1. Do you find it simple to access malaria cases on DHIS2 that were initially reported through the Malaria Connect notification system? Rate from 1 – 5

(1 = Strongly disagree, 2 = disagree, 3 = Neutral, 4 = Agree, 5 = Strongly agree)

___________________

1. Do you find it simple to visualize malaria case data on DHIS2? (Through pivot tables, graphs, and maps) Rate from 1 – 5

(1 = Strongly disagree, 2 = disagree, 3 = Neutral, 4 = Agree, 5 = Strongly agree)

___________________

1. Do you ever find it challenging to visualize malaria case data on DHIS2?

Yes **☐** No **☐** If yes, please indicate the common challenges (select all that apply):

- It is time-consuming **☐**
- It is complicated/complex **☐**
- It is not easy to understand the visualized data **☐**
- Other **☐** Please briefly specify______________

1. Do you find it simple to use the DHIS2 surveillance system to generate or help generate a malaria report/presentation? Rate from 1 – 5

(1 = Strongly disagree, 2 = disagree, 3 = Neutral, 4 = Agree, 5 = Strongly agree)

___________________

**Acceptability**

1. How many days in a week do you log in and use the DHIS2 surveillance system?

- Zero (0) to one (1) day per week **☐**
- Two (2) – four (4) days per week **☐**
- Five (5) days per week **☐**
- More than five (5) days per week **☐**

1. Do you perceive the DHIS2 surveillance system acceptable to you? Rate from 1 – 5 (1 = Strongly disagree, 2 = disagree, 3 = Neutral, 4 = Agree, 5 = Strongly agree)

___________________

1. Are you willing to continue using the DHIS2 surveillance system? Rate from 1 – 5 (1 = Strongly disagree, 2 = disagree, 3 = Neutral, 4 = Agree, 5 = Strongly agree)

___________________

1. Are you willing to continue providing accurate, consistent data into DHIS2? Rate from 1 – 5 (1 = Strongly disagree, 2 = disagree, 3 = Neutral, 4 = Agree, 5 = Strongly agree)

___________________

1. Are you willing to continue providing complete, and timely data into DHIS2? Rate from 1 – 5 (1 = Strongly disagree, 2 = disagree, 3 = Neutral, 4 = Agree, 5 = Strongly agree)

___________________

1. Are there factors that decrease the acceptability of the DHIS2 surveillance system?

Yes **☐** No **☐** If yes, please indicate which factors (select all that apply):

- Work overload **☐**
- Lack of human resources **☐**
- Data costs **☐**
- The complexity of the system **☐**
- Network problems **☐**
- Other **☐** Please briefly specify______________

1. There is adequate dissemination of aggregated malaria case data from Programme managers back to those that notify and input cases? Rate from 1 – 5 (1 = Strongly disagree, 2 = disagree, 3 = Neutral, 4 = Agree, 5 = Strongly agree)

___________________

1. The DHIS2 surveillance system and other users involved in malaria surveillance acknowledge and appreciate my contributions to malaria surveillance? Rate from 1 – 5

(1 = Strongly disagree, 2 = disagree, 3 = Neutral, 4 = Agree, 5 = Strongly agree)

___________________

1. Have you ever made suggestions/comments about improving data reporting into DHIS2?

Yes **☐** No **☐**

If yes, was your suggestion taken? Yes **☐** No **☐**

**This completes the survey. Thank you very much for your participation.**
