# Supplementary material for: Evaluation of the malaria case surveillance system in KwaZulu-Natal Province, South Africa, 2022: a focus on DHIS2
Source: Malar J. 2024 Feb 14;23:47. doi: 10.1186/s12936-024-04873-7 (PMC10865712; doi:10.1186/s12936-024-04873-7)
Supplement: Supplementary file 4 — Additional file 4: Secondary data management flow chart. [file 12936_2024_4873_MOESM4_ESM.docx]

**Additional file 4: Secondary data management flow chart**


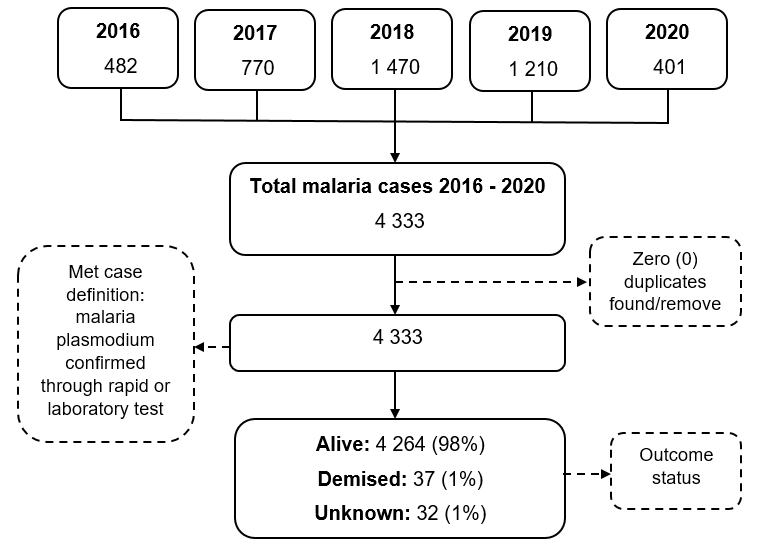


**Figure S1**: Flow chart depicting data management of malaria data retrieved from DHIS2, January 2016 to December 2020
